# Spatio-temporal clustering and meteorological factors influencing HFRS incidence in mainland China, 2004–2021

**DOI:** 10.1017/S0950268825100708

**Published:** 2025-11-03

**Authors:** Hui Jiang, Lijuan Wu, Jin Wei, Huaxiang Rao

**Affiliations:** 1 Department of Biomedical Engineering, Changzhi Medical College, Changzhi, China; 2 School of Public Health, Changzhi Medical College, Changzhi, China; 3 Laboratory of Environmental Factors and Population Health, Shanxi Higher Education Institutions of Science and Technology Innovation Plan Platform, Changzhi, China; 4 Key Laboratory of Environmental Pathogenic Mechanisms and Prevention of Chronic Diseases, Changzhi Medical College, Changzhi, China

**Keywords:** haemorrhagic fever with renal syndrome, meteorological factors, spatial autocorrelation, spatial panel data model, time series decomposition analysis

## Abstract

In this study, HFRS data were obtained from China CDC and ECDC, while monthly meteorological data and GDP were extracted from the National Bureau of Statistics of China website. Descriptive epidemiology, time series decomposition, and spatial autocorrelation analyses were employed to evaluate HFRS incidence patterns. A spatial panel data model was used to estimate the effects of meteorological and socio-economic variables on HFRS incidence. The average annual incidence rate of HFRS was 0.90/100000 in China, compared to 29.3/100000 in Finland. The incidence level in China was comparable to that in Belgium and the EU/EEA (excluding the UK), the high-incidence age group was 45–64 years, which was similar to Finland and the EU/EEA. HFRS in China exhibited marked seasonality. Three north-eastern provinces, Shaanxi, Shandong, and Jiangxi reported higher incidence rates. After adjusting for spatial individual effects and spatial autocorrelation, HFRS incidence was negatively associated with precipitation during the same period, per capita GDP showed no significant effect on HFRS incidence. Continued surveillance and prevention of HFRS remain necessary in China, particularly in Shaanxi. Additional disease prevention and control efforts should be directed towards individuals aged 45–64 years during the high-risk period from October to December.

## Background

Haemorrhagic fever with renal syndrome (HFRS) is a rodent-borne zoonotic infectious disease caused by hantaviruses [1]. These viruses are typically transmitted through aerosolized droplets [2], whereby individuals become infected by inhaling particles contaminated with rodent excreta [1, 3]. In some cases, infection can also occur through bites – either from infected rodents or from mites carrying the virus [4]. Human-to-human transmission of hantaviruses is rare, with only Andes virus known to be transmissible between humans [5]. Enclosed spaces such as barns, garages, and outdoor storage rooms frequented by rodents are common sites of human infection. Engaging in agricultural work, sleeping on the ground, and participating in military exercises are additional risk factors for hantavirus exposure [6]. During cold weather, vole invasions into homes or nesting near residences can further increase the risk of transmission [7]. HFRS has significant implications for human health, as it can lead to fever, haemorrhage, congestion, hypotensive shock, and kidney damage [8].

Approximately 150000 to 200000 cases of HFRS are reported globally each year [8]. Currently, no hantavirus vaccines have been licensed by the World Health Organization [9]. HFRS is the most prevalent zoonosis in Asia and remains common in parts of Europe [2]. China is the country most affected by HFRS. Since it was first identified there in 1931, cases have been reported in 31 provinces, accounting for 70%–90% of reported cases worldwide [10]. In China, HFRS is classified as a Class B notifiable infectious disease [11]. In contrast, HFRS epidemic in parts of Europe is far less severe, with sporadic cases and no large-scale outbreaks ever reported. However, incidence is comparatively high in Northern and Eastern Europe. Vaccination has not yet been widely adopted in Europe; instead, greater reliance is placed on environmental control and surveillance. Only China and South Korea have developed and approved the use of inactivated hantavirus vaccines now [12]. As early as 1994, the Chinese government began offering free vaccinations to individuals aged 16–60 years in high-risk areas [8]. As a result, the number of HFRS cases has dramatically declined in most regions of China [13]. Nevertheless, a resurgence in incidence has been observed in some areas [13]. As a zoonotic disease, HFRS may not cause symptoms in animals after infection, but it can be potentially fatal in humans [14]. The mortality rate for HFRS in Eurasia ranges from 5% to 15% [15]. Although recent advances in vaccination and medical care have reduced mortality, many patients continue to suffer from long-term renal dysfunction or other sequelae following infection [16]. HFRS remains a serious public health concern that should not be overlooked.

Across Asia and Europe, there is currently no systematic comparison of the age distribution of HFRS incidence among countries. Whether the age at peak incidence is consistent across nations or exhibits significant variation remains unclear. Addressing this question is crucial for setting priorities in vaccination strategies, screening high-risk populations, and allocating healthcare resources, and carries both theoretical and practical importance. Therefore, this study conducts a comparative analysis of HFRS in China and selected European countries and evaluates the similarities and differences in incidence characteristics between China and other nations.

Understanding the temporal and spatial distribution of diseases is essential for epidemiological research. Global Moran’s *I*, local Getis-Ord G_i_*, *Kulldorff*’s retrospective scan statistical analysis are the common methods to study the characteristics of spatial distribution [17]. These methods have been widely used in the study of the spatio-temporal dynamic distribution of hand, foot, and mouth disease (HFMD) [18], brucellosis [19], scarlet fever [20], and tuberculosis (TB) [21]. The results showed that these methods were very helpful to identify the high-incidence areas and the dynamic evolution of infectious diseases over the study period.

In HFRS research, some studies focus only on the spatio-temporal distribution within localized regions, and few examine the broader situation across Eurasia [22, 23]; some investigations are limited by short observation period, which do not fully capture the dynamic evolution process of HFRS over time [10, 24]; others include clinical diagnoses, laboratory-confirmed cases, and suspected cases in their study scope [25], which may support evaluation of confirmation rates, but cannot accurately reflect disease incidence. China has a high HFRS burden, with markedly heterogeneous spatial distribution, and pronounced regional disparities. Comprehensive control measures, such as rodent control, vaccination, and sentinel surveillance, have been implemented in high-risk provinces, though strategies differ across regions. Thus, a systematic, long-term analysis of HFRS spatio-temporal dynamics at the provincial level is essential to evaluate the effectiveness of intervention and identify emerging high-risk areas.

Moreover, numerous studies have demonstrated that from an ecological perspective, environmental factors and socio-economic factors critically influence the prevalence of infectious diseases [26, 27]. Climate change is widely recognized as one of the most pressing global threats to human health. However, most methodologies, such as multiple linear regression model and negative binomial regression, seldom incorporate spatial and temporal dimensions. Ignoring heterogeneity across both time and space may exclude important information and lead to divergent conclusions. In our previous study, we employed a spatial panel data model to examine the associations between meteorological factors and the incidence of tuberculosis and scarlet fever. The results indicated that this method reduced error and improved model fit [20, 28].

Therefore, in this study, we applied spatial autocorrelation analysis to investigate the spatio-temporal patterns of HFRS in mainland China from 2004 to 2021, and used a spatial panel data model to estimate the effects of meteorological and socio-economic factors on HFRS incidence.

## Materials and methods

### Ethical statement

This study was approved by the ethics committee of Changzhi Medical College. Patient consent was not required because all the data were collected from the public databases of China and Europe. In addition, no patients’ personal or health information was included in this study.

### Data collection

HFRS data for mainland China from 2004 to 2021 were obtained from the Data Center of the China Public Health Science (Chinese Center for Disease Control and Prevention) (http://www.phsciencedata.cn/Share/). The dataset includes 18 years of monthly case and death counts, along with incidence and mortality rates for 31 administrative regions (23 provinces, 5 autonomous regions, 4 municipalities, and 2 special administrative regions) of mainland China, and annual details for various age groups (excluding Taiwan, Hong Kong, and Macao, because their data were unavailable). Annual population data, monthly meteorological variables (average temperature (MAT, °C), precipitation (MP, mm), total sunshine hours (MSH, h), and relative humidity (RH, %)) for each province, and annual per capita GDP, annual per capita GDP index were extracted from the official website of the National Bureau of Statistics (http://www.stats.gov.cn/tjsj/ndsj/). The per capita GDP index was used to reflect the growth of real per capita GDP after excluding the effects of price changes. The real per capita GDP for each year was derived using the per capita GDP index. HFRS incidence data for Europe (2008–2021) were sourced from European Centre for Disease Prevention and Control (ECDC).

### Statistical analysis methods

#### Seasonal analysis

The seasonal characteristics of HFRS were analysed using time series decomposition. By calculating the ratio of the average number of cases for a given month to the average monthly incidents of 18 × 12 months (2004–2021), the seasonal pattern of HFRS was revealed. When the ratio is close to 1, it indicates no significant seasonal trend [17].

#### Geospatial visualization analysis

Based on annual haemorrhagic fever incidence data, this study utilizes a 1:1000000 map from the National Geospatial Database (http://www.geodata.gov.cn) to show how the incidence has evolved spatially and temporally over 18 years.

#### Three-dimensional (3D) trend analysis

The three-dimensional (3D) trend diagram visually presents the regional incidence in a three-dimensional form. The *x*-axis and *y*-axis represent the longitude and latitude of a specific area, respectively, while the *z*-axis represents the incidence of the specific area [29].

#### Spatial autocorrelation analysis

Spatial autocorrelation refers to the correlation that exists between observations that are spatially adjacent or close to each other. Spatial autocorrelation includes both global spatial autocorrelation and local spatial autocorrelation.

Moran’s *I* is a widely used index for measuring global spatial autocorrelation, and is employed to assess whether the distribution of a variable is random, dispersed, or spatially clustered [30]. The value of Moran’s *I* ranges from −1 to +1, with positive values indicating positive spatial autocorrelation, meaning that similar values tend to cluster in space; negative values indicate negative spatial autocorrelation, meaning that dissimilar values tend to cluster in space; and values close to 0 indicate no significant spatial autocorrelation [31]. The significance of Moran’s *I* was tested by random simulation method. The size of the *p* value reflects the probability that the observed data are spatially random [32]. When the *p* value is less than or equal to 0.05, a positive Moran’s *I* indicates spatial aggregation, and a negative Moran’s *I* indicates spatial discrete distribution. When the *p* value is greater than 0.05, it indicates a spatial random distribution.

LISA maps indicate spatial clustering patterns. High–High (HH) clusters indicate high-incidence provinces, surrounded by other high-incidence provinces, whereas Low–Low clusters indicate low-incidence provinces, surrounded by similarly low-incidence neighbours. High–Low (HL) outliers represent high-incidence areas surrounded by low-incidence areas, while Low–High (LH) outliers indicate low-incidence areas surrounded by high-incidence neighbours [33].

#### Spatial regression analysis

Spatial panel data models are used to account for spatial dependence and allow researchers to capture spatial heterogeneity in the data [34, 35]. The spatial lag panel data model, including the spatial lag fixed effects panel data model (Sar-Panel-FE model) and the spatial lag random effects panel data model (Sar-Panel-RE model), can be written as follows: 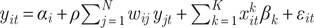 . The spatial error panel data model, including the spatial error fixed effects panel data model (Sem-Panel-FE model) and the spatial error random effects panel data model (Sem-Panel-RE model), can be written as follows: 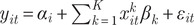 and and 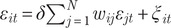 . Where *y_it_* is the HFRS incidence, *α_i_* denotes a spatial specific effect, *x_it_* is the meteorological factor, *k* is the number of factors, *β* is the parameter that expresses the relationship between *y_it_* and *x_it_*, *ε_it_* is the error term. *ρ* and *δ* represent the spatial autocorrelation coefficient.

Model selection was based on statistical criteria, such as goodness of fit, likelihood ratio, and log-likelihood. Given the skewness of HFRS incidence distribution, a natural logarithmic transformation was applied. To handle zero values in the dependent variable, a small constant (0.0001) was added prior to transformation.

#### Statistical software

The disease database was constructed using the Microsoft Excel 2016 application. For the purpose of conducting a comprehensive descriptive epidemiological analysis, the SPSS software (version 22.0, IBM company, New York, USA) was employed. The analysis of spatial distribution, three-dimensional (3D) trend, and spatial autocorrelation of HFRS incidence were utilizing ArcGIS 10.2.2 (ESRI, Redlands, CA, USA). The spatial regression analyses were conducted using Matlab R2016a (Mathworks, Inc., Natick, MA, USA), and the significance level was set as 0.05.

## Results

### Epidemiological characteristics description

**Figure 1.**
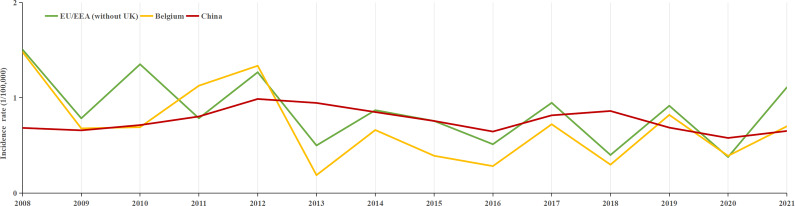
Annual incidence trends of HFRS: Belgium, EU/EEA (without UK), and mainland China from 2008 to 2021.

**Figure 2.**
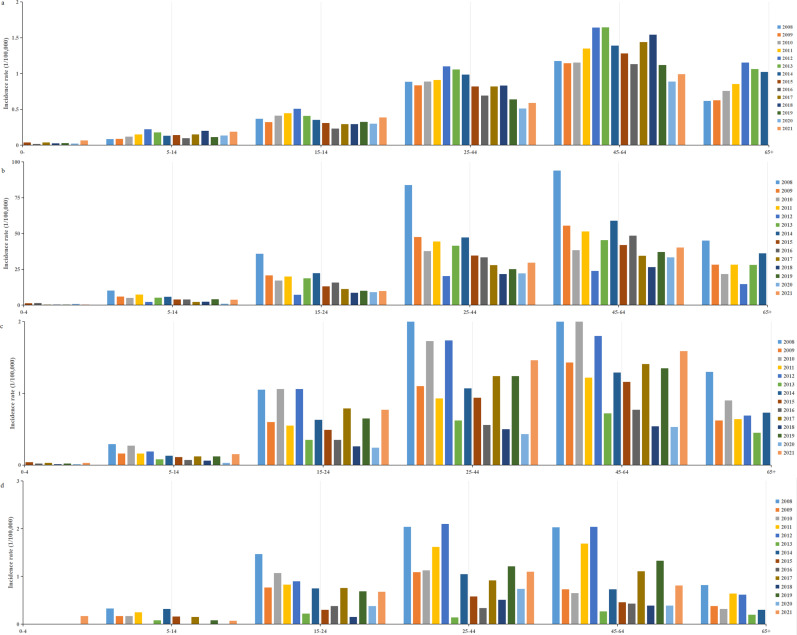
Age-specific distribution of HFRS incidence from 2008 to 2021. (a) China; (b) Finland; (c) EU/EEA (without UK); (d) Belgium.

A total of 217107 HFRS cases were reported between 2004 and 2021 in mainland China, with an average annual incidence rate of 0.8992 per 100000 people. Over this 18-year period, 2006 deaths were attributed to the disease, corresponding to an average annual mortality rate of 0.924 per 100000. Between 2008 and 2021, 145028 individuals were infected with HFRS, with an average annual incidence rate of 0.76 per 100000 people. In comparison, with European countries, Finland had the highest HFRS incidence, with 22296 cases reported between 2008 and 2021, resulting in an average annual rate of 29.3 per 100000. Belgium and the EU/EEA (excluding the UK) exhibited incidence levels comparable to those in China (Figure 1), with average annual rates of 0.69 and 0.84 per 100000, respectively. Finland and the EU/EEA (excluding the UK), like China, reported the highest incidence of HFRS in the 45–64 age group, while Belgium had the highest incidence in the 25–44 age group (Figure 2).

### Temporal distribution

**Figure 3.**
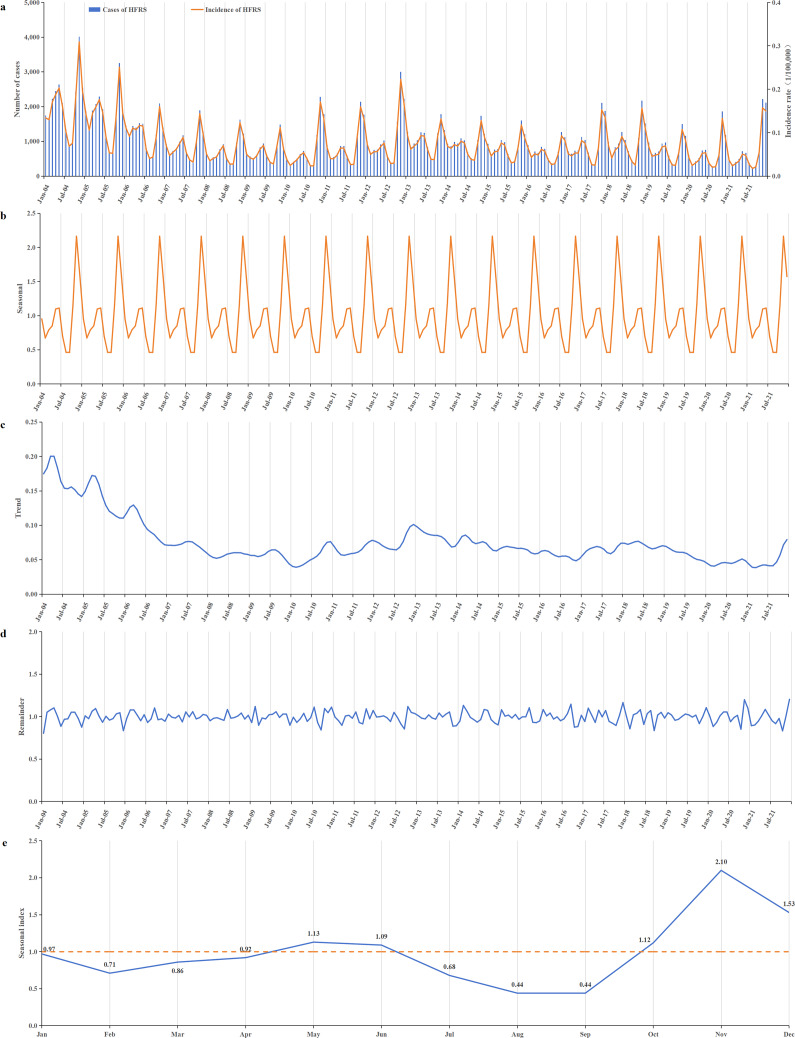
Temporal distribution of reported HFRS incidents from 2004 to 2021. (a) Time series of monthly HFRS incidents; (b) a seasonal trend decomposition of HFRS time series incidents; (c) a long-term trend was decomposed from HFRS time series incidents; (d) the residual data after excluding seasonal and long-term trends; (e) the seasonal index of 12 months ranged from 0.44 to 2.10. A bimodal distribution of epidemic peaks was observed, with peaks occurring in May–June and October–December, and the first peak consistently lower than the second.

During the period from 2004 to 2021, HFRS incidence in China generally showed a downward trend. The incidence was relatively high from 2004 to 2006, with annual rates ranging from 1.155 to 1.926 per 100000. After 2007, the incidence began to decline, ranging from 0.579 to 0.988 per 100000 annually. Apart from a small peak at the end of 2012, the remaining period exhibited relatively stable fluctuations (Figure 3a,c). The lowest incidence was recorded in 2020, at 0.579 per 100000.

HFRS cases were reported every month throughout the year, with distinct seasonal patterns (Figure 3b,e). A bimodal distribution of epidemic peaks was observed, with a smaller, flatter peak occurring in May–June and a more pronounced peak from October to December. The highest seasonal index value of 2.10 occurred in November. Incidence declined during January–February and June–August (Figure 3e).

### Geographic distribution

All provinces in China reported HFRS cases between 2004 and 2021. As shown in the map of average annual incidence, three north-eastern provinces (Heilongjiang, Jilin, and Liaoning), along with Shaanxi, Shandong, and Jiangxi exhibited higher incidence rates. Among these, Heilongjiang and Shaanxi had the highest incidence rates, followed closely by Jilin and Liaoning. In the final 2 years of the study period, the prominence of high incidence in Heilongjiang province decreased notably, whereas Shaanxi continued to report persistently high incidence, remaining the most significantly affected province.

**Figure 4.**
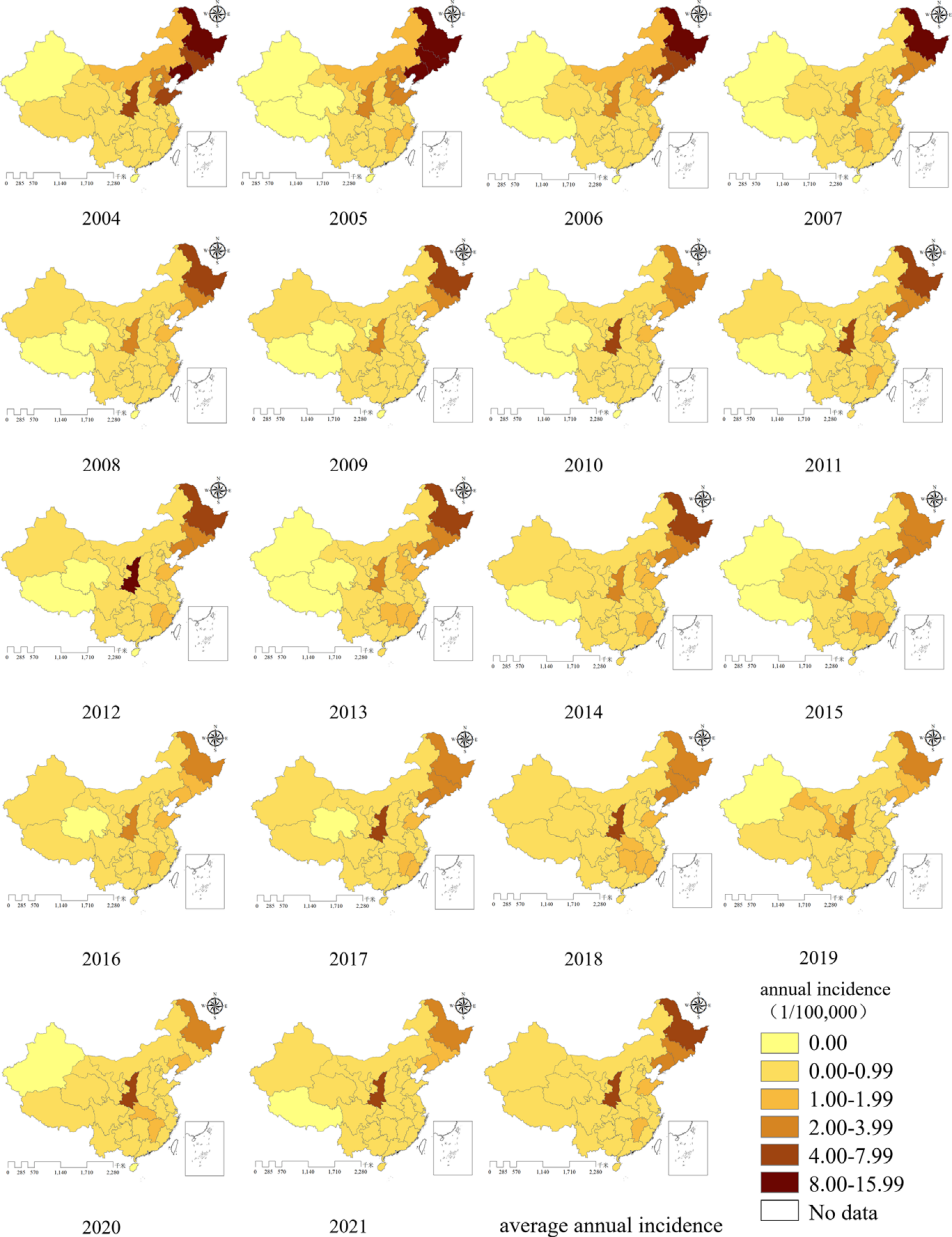
Annual incidence distribution of HFRS in mainland China from 2004 to 2021.

From 2004 to 2021, incidence rates were relatively low in the Xinjiang Uygur Autonomous Region, Tibet Autonomous Region, and Qinghai province. Tibet reported zero incidence for 12 years, Xinjiang and Qinghai for 9 years (Figure 4).

### Three-dimensional (3D) trend

**Figure 5.**
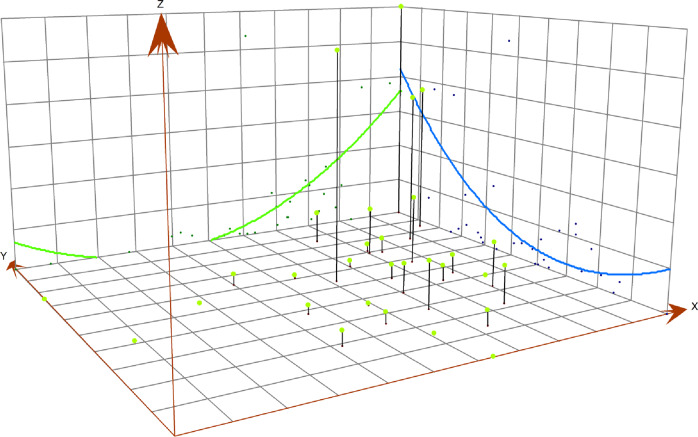
Three-dimensional trend of the average annual reported incidence of HFRS in China, 2004–2021.

HFRS cases were primarily concentrated in the three north-eastern provinces and Shaanxi. The three-dimensional trend analysis showed a similar spatial pattern. In both the north–south and west–east directions, an arch-shaped distribution was observed. In the north–south direction, incidence decreased sharply and then increased slightly. In the west–east direction, a slight decrease was followed by a substantial increase. Overall, incidence was higher in the north than in the south, and lower in the west than in the east (Figure 5).

### Spatial autocorrelation analysis

For the global spatial autocorrelation analysis, provincial administrative regions were used as the units of analysis. The results showed that from 2004 to 2007, the global Moran’s *I* values were all greater than zero (ranging from 0.144 to 0.329), with corresponding Z scores above 1.96 and *p* values below 0.05. These statistically significant results indicate that HFRS prevalence was spatially clustered during this period. However, from 2008 to 2021, all *p* values were greater than 0.05, suggesting that HFRS incidence exhibited a random spatial distribution. Moran’s *I*, Z scores, and *p* values from 2004 to 2021 are presented in Table 1.

**Table 1.** The results of the global Moran’s *I* spatial analysis of annual reported incidence of HFRS in China from 2004 to 2021

Year	Moran’s *I*	V(*I*)	Z score	*p*	Distribution status
2004	0.243	0.010	2.809	0.005	Cluster
2005	0.329	0.010	3.612	0.000	Cluster
2006	0.269	0.009	3.258	0.001	Cluster
2007	0.144	0.007	2.122	0.034	Cluster
2008	0.057	0.010	0.922	0.356	Random
2009	0.095	0.010	1.288	0.198	Random
2010	−0.072	0.008	−0.434	0.664	Random
2011	−0.106	0.008	−0.815	0.415	Random
2012	−0.141	0.007	−1.334	0.182	Random
2013	0.027	0.011	0.589	0.556	Random
2014	0.141	0.010	1.730	0.084	Random
2015	0.010	0.011	0.418	0.676	Random
2016	0.050	0.011	0.799	0.424	Random
2017	−0.085	0.009	−0.554	0.579	Random
2018	0.006	0.010	0.385	0.700	Random
2019	0.042	0.010	0.736	0.462	Random
2020	−0.102	0.006	−0.881	0.378	Random
2021	−0.113	0.004	−1.279	0.201	Random

**Figure 6.**
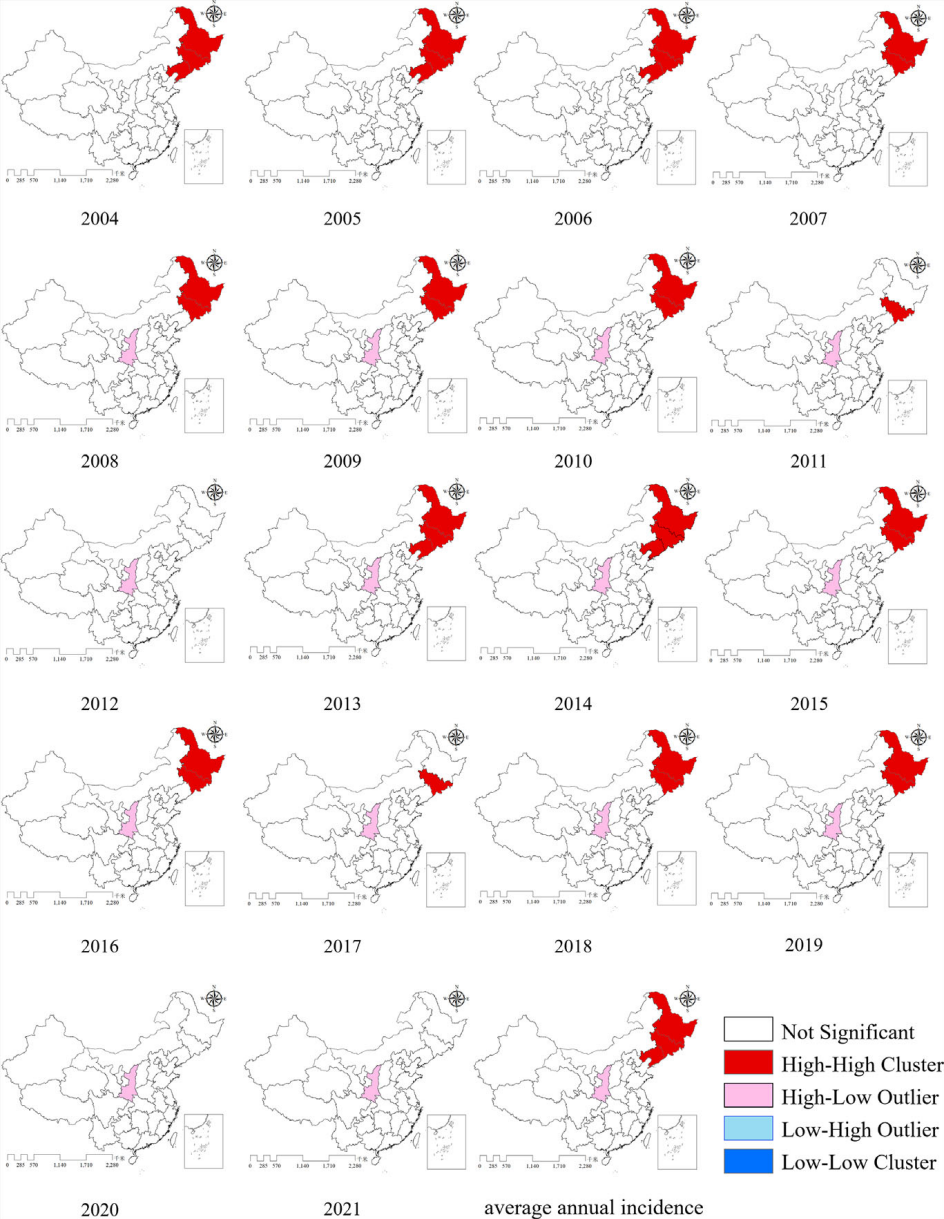
The annual incidence LISA map of HFRS in mainland China from 2004 to 2021.

For the local spatial autocorrelation analysis, the LISA map of average annual incidence showed that the High–High clusters were primarily located in the three north-eastern provinces of China, while a High–Low cluster was observed in Shaanxi province. No statistically significant Low–Low or Low–High clusters were identified. The LISA map further revealed that Jilin province remained a High–High cluster for 15 years, Heilongjiang for 13 years, and Liaoning for 5 years. Shaanxi province was classified as a High–Low area for 14 years (Figure 6).

### Analysis of high-incidence provinces

**Figure 7.**
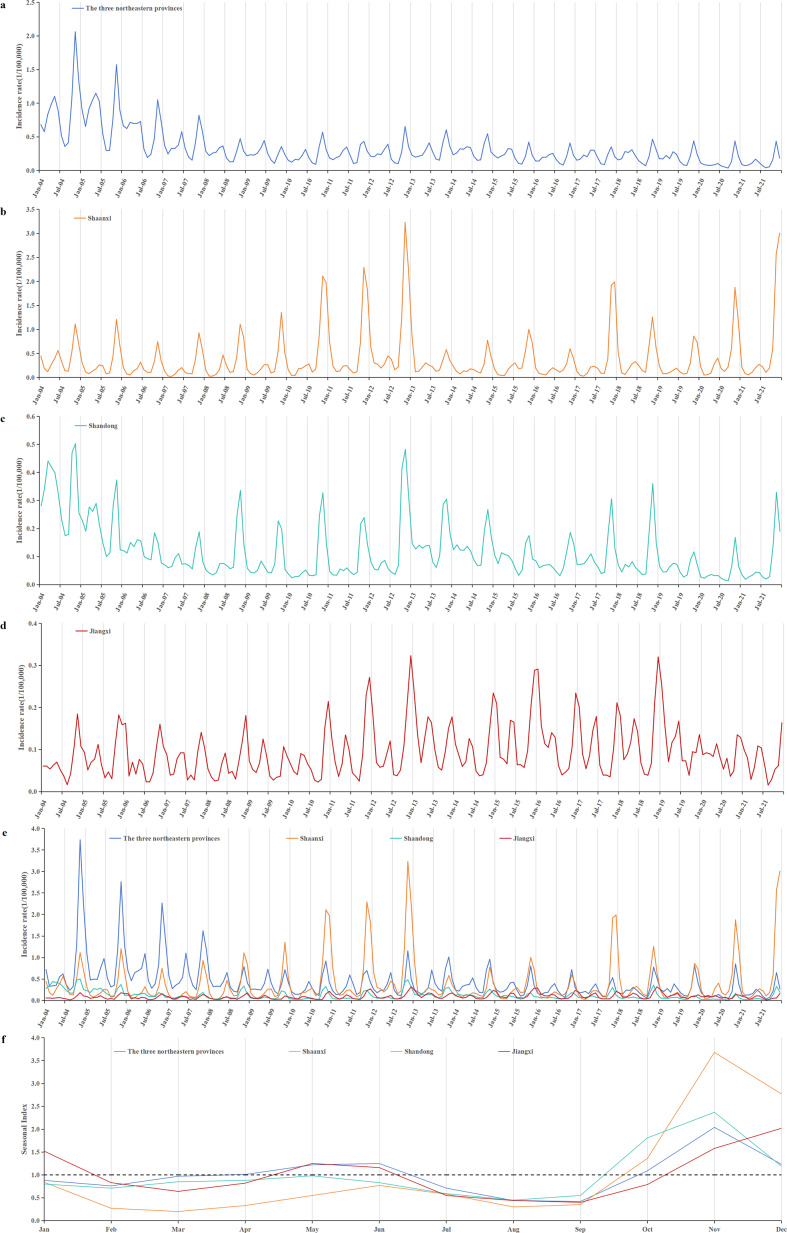
Monthly HFRS incidents of high-incidence provinces. (a) Monthly HFRS incidence of the three north-eastern provinces (including Heilongjiang, Jilin, and Liaoning). (b) Monthly HFRS incidence of Shaanxi. (c) Monthly HFRS incidence of Shandong. (d) Monthly HFRS incidence of Jiangxi. (e) Monthly incidence of HFRS in provinces with high incidence. (f) Comparison of seasonal indices in provinces with high incidence.

Based on the earlier analysis, three north-eastern provinces (Heilongjiang, Jilin, and Liaoning), along with Shaanxi, Shandong, and Jiangxi, exhibited higher incidence rates. The incidence of HFRS in the three north-eastern provinces showed a decreasing trend. After 2008, it reveals a stable fluctuation trend. Meanwhile, the epidemic trend in Shaanxi and Shandong provinces was basically the same. But Shaanxi showed a significant rebound trend in 2021. Similar to Shaanxi and Shandong, there was also a small peak of incidence in Jiangxi at the end of 2012, but the overall trend of Jiangxi was stable and fluctuating. Shaanxi and Shandong experienced only one peak, which occurred from October to December. In contrast, the three north-eastern provinces and Jiangxi had a second peak. The first epidemic peak for these regions occurred in May to June. For Jiangxi province, the second peak was from November to January of the following year, whereas for the three north-eastern provinces, it was from October to December (Figure 7).

### Spatial regression analysis of meteorological and socio-economic effects on HFRS

By regression analysis, we should consider the time lag between the meteorological variables and HFRS incidence because infectious diseases have a certain incubation period. In this study, we used meteorological factors with a 0- to 3-month lag from the HFRS incidence to fit the simple linear model first, and the descriptive statistics for the meteorological variables were shown in Table 2. The results showed that the HFRS incidence and meteorological factors in the same period were found to have the best goodness of fit, but the *R*^2^ and adjusted *R*^2^ were too small (*R*^2^ = 0.0294 and the adjusted *R*^2^ = 0.0288). Building upon our previous research, we applied a spatial panel data model to examine the associations between HFRS incidence and meteorological factors. Among the four types of spatial panel data models tested, the spatial lag fixed effects panel data model (SAR-Panel-FE) provided the best fit for estimating the relationship between HFRS incidence and meteorological factors (Supplementary Table S1).

**Table 2.** Descriptive statistics for meteorological variables in China from January 2004 to December 2021

Meteorological variables	Mean	Standard deviation	Min	Max	Percentiles		
					25	50	75
MP (mm)	76.26	97.59	0.00	1930.00	11.90	44.15	105.88
MAT (°C)	14.46	10.65	−21.10	32.60	7.30	16.30	23.10
RH (%)	64.76	14.95	14.00	95.00	55.00	68.00	76.00
MSH (h)	169.98	67.72	0.00	387.60	121.80	172.60	218.78

Abbreviation: MP, monthly precipitation; MAT, monthly average temperature; RH, relative humidity; MSH, monthly total sunshine hours.

However, in the preceding analysis, only spatial individual effects and spatial autocorrelation were considered, while temporal autocorrelation within provinces was not addressed. To account for this, we included a 1-month lag of incidence as an independent variable in the final regression model. The model demonstrated excellent fit statistics: *R*^2^ = 0.8091, adjusted *R*^2^ = 0.8081, likelihood ratio (LR) = 2219.7802 (*p* < 0.0001), and log-likelihood = −10843.379. The Durbin–Watson statistic (*DW* = 2.0157) indicated no autocorrelation in the error term.

The results revealed considerable variation in background incidence across provinces. The highest background incidence was observed in Shaanxi (1.3701 per 100000), and the lowest in Hainan (0.0027 per 100000) (Table 3). The spatial autocorrelation coefficient was 0.2714, suggesting a spatial spill-over effect. The regression coefficient for the 1-month lagged incidence was 0.3271, indicating that prior incidence significantly influenced current incidence. After adjusting for spatial individual effects and spatial and temporal autocorrelation, HFRS incidence was negatively associated with precipitation (*b* = −0.001016, *p* < 0.001). Specifically, a 10 cm increase in precipitation was associated with a 9.66% decrease in HFRS incidence (Table 4).

**Table 3.** Results for spatial individual effects of each province by using the spatial lag fixed effects panel data model

Province	Intercept term (*α_i_*)	Background incidence (1/100000)	Province	Intercept term (*α_i_*)	Background incidence (1/100000)	Province	Intercept term (*α_i_*)	Background incidence (1/100000)
Shaanxi	0.314861	1.3701	Inner Mongolia	−1.415874	0.2427	Shaanxi	−3.068033	0.0465
Heilongjiang	−0.213675	0.8076	Yunnan	−1.453162	0.2338	Chongqing	−3.664474	0.0256
Liaoning	−0.495514	0.6093	Jiangsu	−1.456809	0.2330	Xinjiang	−3.945082	0.0193
Jilin	−0.569971	0.5655	Guangdong	−1.589487	0.2040	Guangxi	−4.020179	0.0179
Hunan	−0.597929	0.5499	Sichuan	−1.665456	0.1891	Qinghai	−4.167192	0.0155
Shandong	−0.697654	0.4978	Guizhou	−1.707215	0.1814	Tibet	−4.250637	0.0143
Zhejiang	−0.725423	0.4841	Anhui	−1.932561	0.1448	Ningxia	−4.575769	0.0103
Jiangxi	−0.853105	0.4261	Henan	−1.989968	0.1367	Shanghai	−4.860369	0.0077
Hebei	−0.944064	0.3890	Tianjin	−2.454065	0.0859	Hainan	−5.925199	0.0027
Fujian	−1.256813	0.2846	Gansu	−2.549643	0.0781			
Hubei	−1.342471	0.2612	Beijing	−2.997776	0.0499

**Table 4.** Results of the spatial lag fixed effects panel data model for HFRS incidence with meteorological factors

Factors	*b*	*S_b_*	*b′*	95% CIs of coefficients	*t*	*p*
MP (mm)	−0.001016	0.000197	−0.035383	−0.001402, −0.000630	−5.156987	<0.0001
MAT (°C)	−0.000599	0.002311	−0.002324	−0.005131, 0.003932	−0.259271	0.7954
RH (%)	0.003003	0.002052	0.016206	−0.001020, 0.007026	1.463286	0.1434
MSH (h)	−0.000154	0.000446	−0.003568	−0.001028, 0.000720	−0.345389	0.7298
One-month lag ln incidence	0.327068	0.011187	0.326703	0.305137, 0.349000	29.235407	<0.0001
*ρ*	0.271437	0.014997	0.275340	0.242038, 0.300836	18.099610	<0.0001

We also applied a spatial panel data model to assess the effects of socio-economic factors on HFRS incidence. The results indicated that per capita GDP had no statistically significant impact on HFRS incidence (Table 5).

**Table 5.** Results of the spatial lag fixed effects panel data model for HFRS incidence with socio-economic factor

Factors	*b*	*S_b_*	*b′*	95% CIs of coefficients	*t*	*p*
Per capita GDP	0.000007	0.000004	0.071028	−0.000001, 0.000016	1.778441	0.0753
*ρ*	0.213339	0.063760	0.213336	0.088022, 0.338656	3.345959	0.0008

## Discussion

HFRS is characterized by its widespread prevalence, multiple transmission routes, and rapid dissemination, posing a serious threat to public health [36]. It is the most common zoonosis in Asia and is also prevalent in parts of Europe [37–39]. Among European countries, Finland reported the highest incidence of HFRS, with 22296 cases reported between 2008 and 2021, corresponding to an average annual rate of 29.3 per 100000 people. China has the highest number of reported cases in Asia [40], with infections documented across all provinces. During the same period, the incidence rate in China was 0.76 per 100000 people, which is comparable to rates reported in the EU/EEA (excluding the UK) and Belgium.

This study analysed HFRS cases reported in China from 2004 to 2021, based on data obtained from the Data Center of the China Public Health Science. The findings indicate that HFRS incidence in China exhibited a generally declining trend. This trend may be attributed to the implementation of effective prevention and control measures by the Chinese government, increased public awareness and precautionary behaviours, and overall improvements in public health infrastructure. The lowest incidence was recorded in 2020, which may be closely linked to factors related to the COVID-19 pandemic, such as enhanced personal protective measures, widespread, environmental disinfection, reduced outdoor activities, and diminished contact with natural rodent hosts [41].

HFRS affected age groups vary across countries. In the EU, Finland, and China, the disease is most prevalent among individuals aged 45–64 years, while Belgium exhibits higher incidence in the 25–44 age group. These findings are generally consistent with previous domestic research [42–44]. This pattern may be due to increased occupational exposure, in these age group, as individuals engaged in more productive labour, or fieldwork are more likely, to encounter rodent hosts [45, 46].

HFRS cases were reported every month throughout the year, exhibiting clear seasonal characteristics. A bimodal distribution pattern was observed, with a steady smaller peak in May–June and a more pronounced peak from October to December. However, analysis of provinces with high incidence revealed that Shaanxi and Shandong experienced only a single peak, occurring from October to December. In contrast, Jiangxi exhibited a second peak from November to January, consistent with previous findings [10, 47]. This pattern may be influenced by the climate of Jiangxi province. Differences in peak timing across meteorological zones were also observed, aligning with the results reported by Zou [48].

All provinces in China reported HFRS cases from 2004 to 2021. The incidence rate was higher in the north than in the south, and lower in the west than in the east [26]. Three north-eastern provinces (Heilongjiang, Jilin, and Liaoning), along with Shaanxi, Shandong, and Jiangxi, exhibited higher incidence rates, which aligns well with previous domestic research findings [8, 48]. From 2004 to 2007, the prevalence of HFRS was spatially clustered; however, from 2008 to 2021, it exhibited a random spatial distribution. High–High clusters were mainly concentrated in the three north-eastern provinces, while a High–Low cluster was consistently observed in Shaanxi province. No significant Low–Low and Low–High clusters was identified. The LISA map also revealed that during the study period, Jilin province was classified as a High–High cluster for 15 years, Heilongjiang for 13 years, and Liaoning for 5 years. Shaanxi province was categorized as a High–Low cluster for 14 years. These patterns are consistent with the ‘spill-over’ effect associated with rodent hosts. HFRS results from the spill over of hantaviruses from rodents hosts to humans, with no human-to-human transmission via contact. A stable transmission cycle has been established among rodent hosts in nature, and under suitable conditions, spill over to humans can occur, leading to outbreaks [49].

The regression analysis showed that the spatial autocorrelation coefficient was 0.2714, indicating that HFRS incidence in a given province increased by 31.18% when the incidence in adjacent provinces increased by 1.72 times. The coefficient for the 1-month lagged incidence was 0.3271, suggesting that current HFRS incidence increased by 38.69% when the previous month’s incidence rose by 1.72 times holding other influencing factors constant. After adjusting for spatial individual effects and spatial and temporal autocorrelation, same-period precipitation emerged as the primary meteorological factor influencing HFRS incidence. HFRS is primarily transmitted through human–rodent contact. Increased precipitation floods rodent burrows, reducing local rodent density and thereby decreasing the frequency of human–rodent encounters, which lowers HFRS incidence. Our findings were similar with those from Shandong [50, 51], Guangzhou [52], and low-lying areas of China [53], and inconsistent with the result from the warm temperate and sub-tropical zones of China [36]. In Luo’s study, distributed lag non-linear models (DLNMs) were used to assess the effects of temperature, precipitation, and relative humidity on HFRS incidence. In contrast, our study employed a spatial panel data model that additionally incorporated sunshine duration among the meteorological variables. These discrepancies suggest that the influence of meteorological factors on infectious disease incidence is sensitive to both variable selection and analytical framework, warranting further systematic investigation.

Per capita GDP showed no significant association with HFRS incidence, which is consistent with the findings from Shandong [51]. This result implies that per capita GDP may not directly influence transmission dynamics, though unmeasured mediators, such as public health investment or living conditions, may play a role but were not included in the current model.

Building on the earlier findings, in the high-incidence provinces (Heilongjiang, Jilin, Liaoning, Shaanxi, Shandong, and Jiangxi), vaccination coverage should be reinforced among individuals aged 45–64 years. In parallel, a meteorological early-warning system should be established. If precipitation during the same period is forecasted to decline, county-level CDCs and hospitals will be alerted 1 month in advance.

This study had several limitations. First, only per-capita GDP and four meteorological variables were included as explanatory factors; however, other unmeasured determinants, such as land-use changes, rodent density, vaccination coverage, and public health interventions, may also influence HFRS incidence and warrant further investigation. Second, interactions among meteorological variables were not considered, which may have obscured their joint effects on HFRS incidence. Third, this was an ecological study, which examined associations at the group level, therefore, individual-level effects, such as the probability of infection could not be assessed.

## Conclusions

HFRS remains a public health concern and requires sustained attention and prevention efforts, particularly in Shaanxi province. Additional control measures should be directed towards individuals aged 45–64 years during the high-incidence period from October to December. Regions with low precipitation tended to report higher HFRS incidence, indicating the need for strengthened prevention strategies in these areas. Future research should further explore additional influencing factors to support evidence-based policymaking and enhance disease control strategies.

## Supporting information

10.1017/S0950268825100708.sm001 Jiang et al. supplementary material Jiang et al. supplementary material

## Data Availability

The dataset analysed during this study was collected from public database of China and Europe, and is publicly available online.

## Supplementary material

The supplementary material for this article can be found at http://doi.org/10.1017/S0950268825100708.
