# Supplementary material for: Spatio-temporal clustering and meteorological factors influencing HFRS incidence in mainland China, 2004–2021
Source: Epidemiol Infect. 2025 Nov 3;153:e130. doi: 10.1017/S0950268825100708 (PMC12641306; doi:10.1017/S0950268825100708)
Supplement: Jiang et al. supplementary material [file S0950268825100708sup001.docx]

**Table S1** Four kinds of spatial panel data models for HFRS incidence with meteorological factors

| **Factors** | **Sar-Panel-FE** | | |  | **Sar-Panel-RE** | | |  | **Sem-Panel-FE** | | |  | **Sem-Panel-RE** | | |
| --- | --- | --- | --- | --- | --- | --- | --- | --- | --- | --- | --- | --- | --- | --- | --- |
|  | ***b*** | ***t*-Value** | ***P*-Value** |  | ***b*** | ***t*-Value** | ***P*-Value** |  | ***b*** | ***t*-Value** | ***P*-Value** |  | ***b*** | ***t*-Value** | ***P*-Value** |
| Constant |  |  |  |  | -2.962916 | -6.531098 | <0.0001 |  |  |  |  |  | -4.524229 | -9.649839 | <0.0001 |
| MP (mm) | -0.000079 | -0.382140 | 0.7024 |  | -0.000079 | -0.380515 | 0.7036 |  | -0.000130 | -0.588847 | 0.5560 |  | -0.000130 | -0.587517 | 0.5569 |
| MAT (℃) | -0.014828 | -6.107198 | <0.0001 |  | -0.014832 | -6.097778 | <0.0001 |  | -0.023446 | -7.566463 | <0.0001 |  | -0.023523 | -7.546035 | <0.0001 |
| RH (%) | -0.001526 | -0.708214 | 0.4788 |  | -0.001499 | -0.694606 | 0.4873 |  | -0.000971 | -0.407573 | 0.6834 |  | -0.000851 | -0.356248 | 0.7217 |
| MSH (h) | -0.000352 | -0.753324 | 0.4513 |  | -0.000363 | -0.775018 | 0.4383 |  | -0.000129 | -0.252069 | 0.8010 |  | -0.000122 | -0.237772 | 0.8121 |
| *ρ* | 0.344326 | 23.199721 | <0.0001 |  | 0.343332 | 23.128090 | <0.0001 |  |  |  |  |  |  |  |  |
| *δ* |  |  |  |  |  |  |  |  | 0.344999 | 23.243005 | <0.0001 |  | 0.350222 | 23.641378 | <0.0001 |
| *R*-squared | 0.7889 |  |  |  | 0.7879 |  |  |  | 0.7661 |  |  |  | 0.7882 |  |  |
| Adjusted *R*-squared | 0.7878 |  |  |  | 0.7877 |  |  |  | 0.7650 |  |  |  | 0.7880 |  |  |
| Log likelihood | -11270.925 |  |  |  | -11388.128 |  |  |  | -11270.575 |  |  |  | -11390.419 |  |  |
| *LR* | 9729.3422 |  | <0.0001 |  | 9494.9355 |  | <0.0001 |  | 9730.0321 |  | <0.0001 |  | 9490.3446 |  | <0.0001 |
| Durbin-Watson statistic | 2.0314 |  |  |  | 2.0314 |  |  |  | 1.8531 |  |  |  | 2.0336 |  |  |
